# Influence of Micronutrient Intake, Sociodemographic, and Behavioral Factors on Periodontal Status of Adults Assisted by a Public Health Care System in Brazil: A Cross-Sectional Multivariate Analysis

**DOI:** 10.3390/nu13030973

**Published:** 2021-03-17

**Authors:** Patrícia Daniela Costa, Juliana Cristina Reis Canaan, Paula Midori Castelo, Douglas Campideli Fonseca, Stela Márcia Pereira-Dourado, Ramiro Mendonça Murata, Vanessa Pardi, Luciano José Pereira

**Affiliations:** 1 Health Sciences Faculty, Universidade Federal de Lavras (UFLA), 37200-900 Lavras, Minas Gerais, Brazil; patriciadaniela.costa@yahoo.com.br (P.D.C.); reisjuliana@yahoo.com.br (J.C.R.C.); stelapereira@ufla.br (S.M.P.-D.); 2 Department of Pharmaceutical Sciences, Universidade Federal de São Paulo (UNIFESP), 09913-030 Diadema, São Paulo, Brazil; paula.castelo@unifesp.br; 3 Dental School, Centro Universitário de Lavras (UNILAVRAS), 37200-000 Lavras, Minas Gerais, Brazil; douglas.perio@gmail.com; 4 Department of Foundational Sciences, School of Dental Medicine, East Carolina University (ECU), Greenville, NC 27834, USA; muratar16@ecu.edu

**Keywords:** periodontal diseases, public health, nutritional research, micronutrients

## Abstract

The lack of access to a balanced diet, rich in vitamins and minerals, can predispose people to inflammatory diseases such as obesity, diabetes mellitus, and periodontitis. We aimed to evaluate the relationship between micronutrient intake, sociodemographic behavioral characteristics, and periodontal health in adults assisted by a public health care system. Participants (*n* = 450) answered a food frequency questionnaire and were submitted to anthropometric and oral clinical examinations. Principal component analysis was used to summarize the number of components emerging from 17-micronutrient intake. Subsequently, cluster analysis was employed. The prevalence of at least one periodontal pocket ≥ 4 mm was 67.4%. Three clusters were identified according to periodontal status. Cluster 1 “poor periodontal status” was characterized by older individuals (*n* = 202; 85% females) with poor periodontal status, lower education level, mainly smokers with non-transmissible chronic diseases (NTCD), with lower energy, omega-3, fiber, Zn, K, Cu, and vitamin C intake. Cluster 3 “healthy periodontal status” included younger individuals (*n* = 54) with the healthiest periodontal status, a higher education level, without NTCD, and with higher energy, omega-3, fiber, Zn, calcium, retinol, and riboflavin intake. Cluster 2 was labeled as “intermediate periodontal status”. Micronutrient ingestion was associated with periodontal status and may be considered in health promotion actions for low-income populations.

## 1. Introduction

Periodontal diseases represent a set of inflammatory conditions in the supporting tissues of the teeth, initiated by the presence of a dysbiotic biofilm [1,2] in the favor of pathogenic bacteria such as *Porphyromonas gingivalis, Prevotella intermedia, Aggregactibacter actinomycetemcomitans,* and *Fusobacterium nucleatum* [3]. Bacterial products initiate a local inflammatory response in the gingival tissue, releasing interleukins, prostaglandins, and other cytokines that activate osteoclasts [4,5,6]. Such activation leads to the resorption of the alveolar bone with a consequent deepening of the gingival sulcus, generating mobility and in some cases even tooth loss [7]. The high prevalence of periodontitis constitutes an important public health problem [8,9,10]. When untreated, periodontitis can lead to a loss of masticatory function [11], aesthetic damage, and impaired social interaction and quality of life [12].

As the progression of periodontitis depends on the activity of the host’s immune system, nutritional factors have been highlighted as sources of interference and modulation. A deficient diet, lacking fruits and vegetables, and therefore poor in micronutrients (such as vitamins and minerals) favors the exacerbation of the periodontal inflammatory response [13]. Micronutrients are essential for maintaining general health, being necessary for the synthesis of a variety of structural biomolecules and also for immune functions [14]. The identification of a nutritional profile associated with better periodontal health can generate inexpensive and safe methods for the prevention and treatment of pathological conditions of the periodontium [15] at the population level.

A low-carbohydrate diet, rich in omega-3 fatty acids, vitamins C and D, and fiber, can significantly reduce gum and periodontal inflammation [16,17,18,19,20,21,22]. However, few studies have been conducted on populations from upper-middle-income countries, where social and nutritional issues are different from the context of developed countries. In addition, Brazil has a universal, free, and public health care system that greatly modifies the context of assessment in relation to previous studies [23,24]. The family health strategy (FHS) is a model of primary health care (PHC) that uses a multidisciplinary approach. The services offered include medical consultations, dental care, preventive exams, and home visits [25,26,27]. About 65% of households in Brazil are registered at the FHS, with the majority of this population having a low level of education and low income [27,28,29]. The FHS represents the gateway to Brazilian public health care. Therefore, understanding the social determinants of the health–disease process in this population allows for a wide range of actions directed to health care, prevention, and promotion [30].

The present study aimed to examine the relationship between micronutrient intake, sociobehavioral factors, chronic diseases, and periodontal status in adults assisted by the public primary health care system. The findings of the present study may guide low-cost and population-based preventive measures for the public health system, considering the characteristics of low-income populations.

## 2. Materials and Methods

### 2.1. Study Sample

The study was carried out in the units of the FHS in the city of Lavras (approximately 110,000 citizens, demographic density 163.26 inhabit/km²), latitude 21°14′ 43 south and at a longitude 44°59′ 59 west, in the state of Minas Gerais, Brazil during the years of 2019–2020. The last human development index (HDI, 2010) of Lavras is 0.782, which places this municipality in the high range of the HDI (0.700–0.799). We decided to study a population that is served by FHS because this strategy covers the majority of households in Brazil and represents the main input to the Brazilian public health care system. This study initiated on March 2019 and lasted until August 2020. The present research protocol was approved by the Human Research Ethics Committee of the Federal University of Lavras (COEP/UFLA, Minas Gerais, Brazil—Protocol Number: 85767618.1.0000.5148). All procedures were in accordance with the Ministry of Health resolution 466/12 from the Brazilian government. We followed the Strengthening the Reporting of Observational Studies in Epidemiology (STROBE) statement checklist for cross-sectional studies. The research participants read and signed the free and informed consent form (ICF) and all doubts were solved before any procedure was performed.

**Figure 1:**
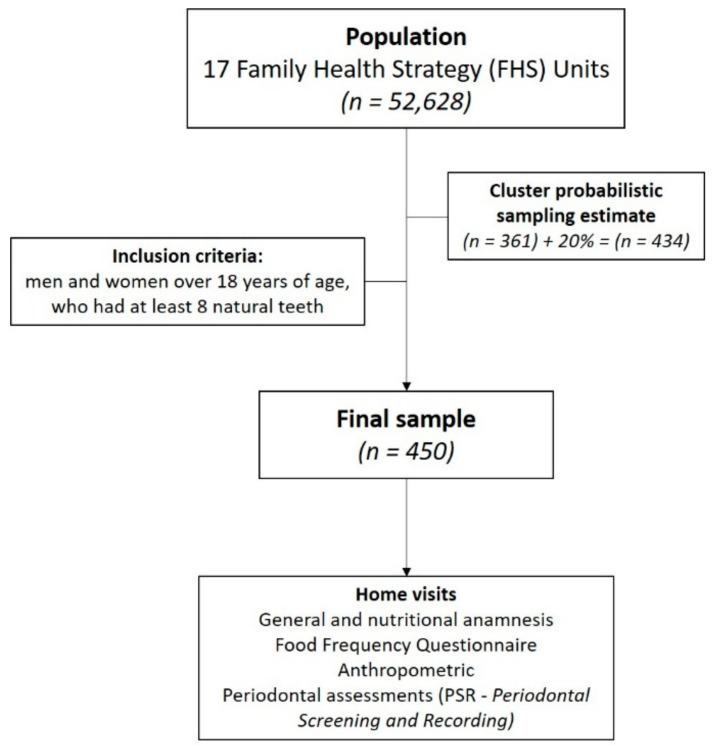
Flowchart of the study design and sample selection.

The participants were selected randomly by means of probabilistic sampling by clusters, proportionally among the 17 different city health units, within people registered with the FHS at the time of the project’s start (52,628 individuals). The selection was systematic, based on patient records, in accordance with the inclusion criteria, comprising all micro areas of each health agent in each unit. The inclusion criteria for study participants were: men and women over 18 years of age, who had at least 8 natural teeth, and who agreed to participate voluntarily in the research project. People with mental health disorders, under orthodontic treatment, and pregnant women were excluded. The sample calculation was performed using the estimated prevalence of periodontitis [31], with precision of the absolute estimate of 5% and a significance level of 5% [32], resulting in a sample of 361 individuals. Due to the possibility of losses and the variability in the number of individuals belonging to each coverage region, this calculation was increased by 20%, totaling the minimum number of 434 individuals. It was then decided to evaluate 450 individuals (Figure 1).

### 2.2. Data Collection

Data collections related to nutritional variables and the periodontal status of the population were carried out in a cross-sectional design, through home visits. In each visit, general and nutritional interviews were carried out, also applying the food frequency questionnaire [33], followed by anthropometric [34] and periodontal assessments [35]. The same nutritionist professional, a member of the team, collected nutritional data with the aid of an album containing photos of portion sizes (developed to facilitate volunteers to visualize their food portions). The amount and types of food intake reported by each volunteer were screened for the amount of micronutrients such as omega-3 (N-3), omega-6 (N-6), iron (Fe), copper (Cu), magnesium (Mg), manganese (Mn), phosphorus (P), sodium (Na), potassium (K), zinc (Zn), calcium (Ca), dietary fiber, vitamin C, vitamin A (retinol), thiamine, riboflavin, pyridoxine, and niacin, as well as macronutrients, carbohydrate, proteins, and lipids.

Anthropometric measurements were performed according to the technical standard of the food and nutrition surveillance system (SISVAN) [34]. The body mass index (BMI = weight (Kg)/(height (m))²) was calculated [36]. To assess the waist circumference, data were collected and categorized according to the risk for metabolic complications (men > 94 cm and women > 80 cm) [37].

The periodontal clinical examination was performed on all volunteers by a single examiner, properly trained and calibrated, in the room with best natural lighting. The individual remained seated in a chair and the examiner, wearing proper personal protective equipment, was standing beside the volunteer. A Williams-type periodontal probe with gradations of 1, 2, 3, 5, 7, 8, 9, and 10 mm, and a sterile mouth mirror (Trinity Indústria e Comércio Ltda., São Paulo, Brazil) were used.

Periodontal evaluation was performed using PSR (periodontal screening and recording) [35,38]. All teeth were evaluated (with the exception of third molars). The mouth was split into sextants and each of them was classified using an ordinal scale starting from healthy (score 0), bleeding on probing (score 1), presence of calculus (score 2), probing depth of 4–5 mm (score 3), and probing depth ≥6 mm (score 4). If all teeth of a given sextant were absent (toothless), no observations were recorded for that sextant. We used the worst sextant (highest score) to determine patient’s periodontal status.

Variables including sex, age, educational level, smoking habits, family income, and frequency of physical activity, as well as the presence of non-transmissible chronic diseases such as diabetes mellitus, hypertension, and dyslipidemia were obtained through interviews. The educational level was categorized as up to 8 years of study or more. Smoking was classified as non-smoker or smoker. Alcohol consumption was dichotomized at a frequency greater than or equal to twice a week. Family income was dichotomized at up to two or more minimum wages (about US$ 500).

### 2.3. Statistics

Statistical analysis was performed using SPSS v26.0 software (IBM SPSS Statistics for Windows, v26.0. Armonk, NY, USA: IBM Corp) considering an alpha level of 5% by an applied statistics spec. Principal component analysis (PCA) was used to estimate the number of components emerging from micronutrients intake (Omega-3, total cholesterol, fiber, calcium, Mg, Mn, P, Fe, Na, K, Cu, Zn, retinol, thiamine, riboflavin, pyridoxine, niacin, and vitamin C) after Z-score transformation. First, the correlation matrix of the standardized variables was examined, and the number of components to retain was based on eigenvalues, total of explained variance, and scree plot examination. As the variables showed moderate correlations, an oblimin rotation was performed. The overall Kaiser–Meyer–Olkin (KMO) measure and Bartlett’s test of sphericity were examined, which are required for a good principal component analysis.

Furthermore, K-means cluster analysis was performed to identify groups of participants with similar nutritional intake, chronic diseases (diabetes mellitus, hypertension, hypercholesterolemia, hypertriglyceridemia, hypothyroidism, liver steatosis, cardiopathy, cancer history, depression), anthropometric characteristics and, periodontal status. The analysis included the following variables: age, sex, BMI, physical activity, chronic diseases, smoking, alcohol consumption, income, formal education, dental treatment in the last year, and component loadings generated from PCA summarizing micronutrients data. The final number of clusters was based on the interpretability and reliability of the cluster solution; the silhouette coefficient and the differences between clusters assessed by One-way ANOVA testing was used for clustering validation.

## 3. Results

The mean age of participants was approximately 50 years old. The prevalence of at least one periodontal pocket ≥ 4 mm was 67.4% (PSR scores 3 and 4). In general, the sample comprised of overweight (mean BMI = 28.5), sedentary (66.25%), and non-smoking (75.9%) participants. The prevalence of diabetes mellitus was 28.2%, whereas high blood pressure affected 50% of the individuals. Alcohol consumption was more frequent among men (20%). Around 40% of the sample had undergone dental treatment within the last year, and 65.75% of them had a low family income (Table 1). Protein (46 g/day for women and 56 g/day for men) intake was above recommended levels in accordance with dietary reference [39]. Fiber intake was below the recommended level for adults (25 g/day for women and 38 g/day for men), as was as omega-3 (1.1 mg/day and 1.6 mg/day for women and men) and zinc (8 mg/day for women and 11 mg/day for men) (Table 1).

A principal component analysis (PCA) with oblimin rotation was run to identify micronutrient dietary patterns within the study population. The suitability of PCA was assessed prior to analysis; the overall Kaiser–Meyer–Olkin (KMO) measure was 0.813, considered to be “meritorious” according to Kaiser (1974). Bartlett’s test of sphericity was statistically significant (*p* < 0.0001), indicating that the data was likely factorizable.

**Figure 2:**
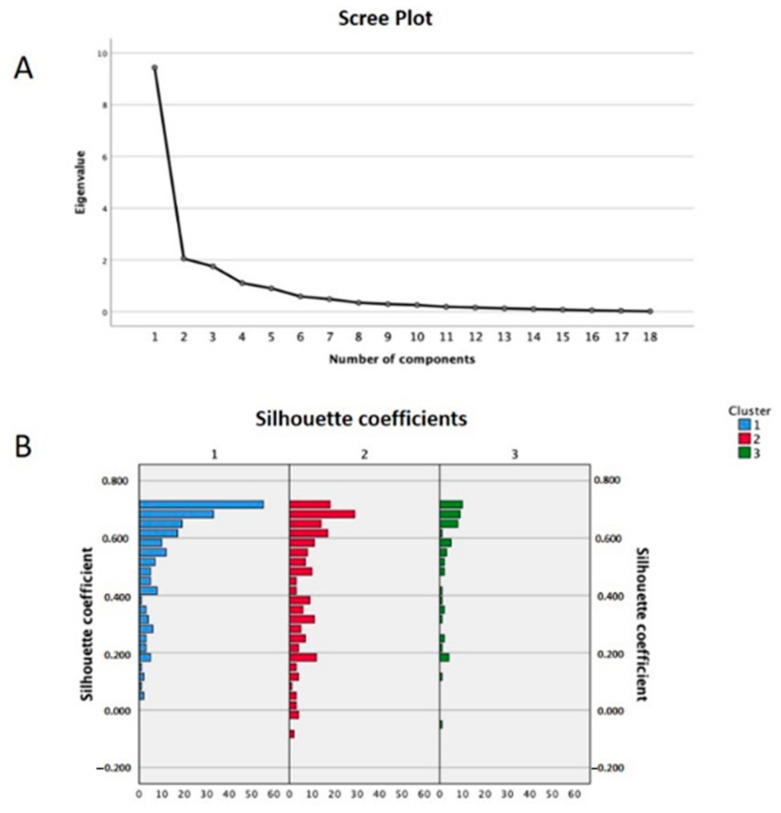
(**A**) Scree plot with the inflection point at component 3 used to determine the number of components to be retained. (**B**) Silhouette plot used to examine internal validity of the cluster solution. The X-axis shows the number of cases for each silhouette coefficient shown in the Y-axis.

After oblimin rotation of the factors, PCA revealed three components that explained 74% of the total variance, as confirmed by visual inspection of the scree plot below (Figure 2A) that was used to identify the number of factors to be retained. As such, three components met the interpretability criterion and were retained, as observed in Table 2.

For interpretation purposes, by examining Table 2 it can be assumed that for component 1, the higher the component, the higher the scores of N-3, cholesterol, fiber, Mg, Mn P, Fe, K, and Zn intake; for component 2, the higher the component, the lower the fiber, Mg, Mn, K, Cu, and vitamin C intake (as the coefficient loadings of component 2 are negative). Finally, for component 3, the higher the component, the higher the calcium, P, retinol, and riboflavin intake.

Furthermore, K-means cluster analysis included the variables: age, sex, BMI, physical activity, chronic diseases, smoking, alcohol consumption, income, schooling, dental treatment in the last year, and the three component loadings generated from PCA. Analysis identified three reliable and meaningful clusters (Table 3), with an average silhouette width of 0.53 (Figure 2B). The plot indicates that there is a good structure to the clusters, with most observations seeming to belong to the cluster that they were in.

Clusters varied significantly according to periodontal status, age, sex, energy, micronutrient intake, smoking, alcohol consumption, educational level, and chronic diseases: hypertension, hypercholesterolemia, and liver steatosis. Graphical Abstract shows the taxonomy description of the three clusters: cluster 1 (labeled “poor periodontal status”, *n* = 202) was characterized by older participants with a higher frequency of chronic diseases and smoking habits, lower energy intake, lower educational level, and being 85% females. This cluster also showed a higher mean for component 2, being characterized by the lower intake of fiber, K, Cu, and vitamin C (indicating a low consumption of micronutrients of this component, since coefficient loadings are negative, see Table 2); besides, the cluster showed a lower mean for component 1, reinforcing the lower N-3, fiber, and Zn intake.

Cluster 3 (labeled “healthy periodontal status”, *n* = 54) was characterized by younger participants with a lower frequency of periodontal disease, a proportionally lower frequency of females (65%), higher energy intake, lower frequency of chronic diseases, higher alcohol consumption, higher educational level, and higher means for components 1 and 3, being characterized by higher N-3, fiber, Zn, calcium, retinol, and riboflavin consumption. Finally, cluster 2 (labeled “intermediate periodontal status”, *n* = 194) was the contrast, being characterized by the low frequency of liver steatosis and moderate periodontal disease.

## 4. Discussion

The results of the present study highlighted the association between micronutrient intake, mainly of zinc, fibers, and omega-3, and periodontal status. Higher consumption of these micronutrients, together with higher energy intake, higher education level, and lower frequency of NTCD, was associated with better periodontal status. On the other hand, the lower energy intake and low consumption of omega-3, fibers, zinc, lower education level, smoking habits, and the presence of NTCD were associated with worse periodontal status. Other micronutrients such as iron, calcium, retinol, and riboflavin were also consumed more by individuals of the “healthy periodontal status” cluster, while those with the worst periodontal status showed a lower consumption of vitamin C, copper, potassium, and magnesium.

Regarding the consumption of zinc, our results corroborate a previous study in which serum levels of this metal were significantly reduced in patients with periodontitis compared to healthy controls [14]. Zinc, like copper and magnesium, plays an important role in the immune response, as well as in preventing oxidative stress. It helps to modulate the release of cytokines, induces the proliferation of CD 8+ T cells (cytotoxic T lymphocytes), assists in maintaining mucosal integrity, and plays an important role in the growth and differentiation of immune cells, as well as in the activation of T lymphocytes and Th1 response (cell-mediated immunity) [40]. The deficiency of this micronutrient impairs the function of neutrophils and macrophages, favors oxidative processes, and reduces the regenerative capacity [41]. Zinc is a cofactor in more than 300 enzymes and transcription factors, acting mainly as a structural stabilizer [42,43,44]. Zinc is essential in the repair of injuries, bone mineralization, blood clotting, and many other homeostatic processes linked in some way to periodontal health [45]. The minimum daily consumption recommendation is told to be 8 mg/day for women and 11 mg/day for men [39], but some authors indicate even higher values of 15 mg/day [46] (a much higher value in comparison to the average intake found in the present sample). Finally, zinc is involved in phosphorylation of insulin receptors [47], and its deficiency leads to hyperglycemia and potentially to type 2 diabetes mellitus due to insulin resistance [48]. In a bidirectional way, diabetes can also alter zinc homeostasis [49] due to hyperzincuria and reduced intestinal zinc absorption [48,50]. This fact was observed in our study in which individuals with a worse periodontal status had a higher frequency of chronic diseases, such as diabetes.

Animal studies have shown that an increased intake of dietary fiber can reduce the progression of periodontal disease [51,52,53,54]. In humans, a study involving 6052 adults also reported that low dietary fiber consumption was associated with worse periodontal status [55]. The probable mechanism arises from the fact that fibers form a gelatinous layer in the intestine that acts as a barrier, making it difficult for pathogens to penetrate. Additionally, the fermentation of fibers in the intestine alters the microbial profile and increases the generation of short-chain fatty acids (SCFA) that stimulate the production of immunoglobulins A (IgA), improving the systemic immune response [56]. Thus, fiber modulates the expression of inflammatory mediators, such as cyclooxygenase-2 (COX-2), and interleukins (IL-1-α, IL-18), reduces the expression receptor activator of the nuclear factor κB ligand (RANK-L), and increases the release of osteoprotegerin (OPG), avoiding alveolar bone loss [17,52]. The physical–chemical characteristics of the fibers differ due to their source and, therefore, promote different local and systemic effects on the human organism. Differences in water retention capacity, viscosity, fermentation, and adsorption, among others, are responsible for metabolic implications (systemic effects), as well as in the gastrointestinal tract (local effects), which are dependent on the type and amount of food consumed [57].

The health benefits of consuming omega-3 are widely known, with great evidence of an immunomodulatory effect in inflammatory status [58]. A systematic review by our group investigated the relationship between plasma concentrations of eicosapentaenoic acid (EPA), docosahexaenoic acid (DHA), and/or arachidonic acid (AA) and periodontal disease. Individuals with higher blood levels of omega-3 from a diet rich in these fatty acids had better periodontal status [59]. When the n3/n6 ratio is increased by the intake of omega-3 fatty acids (DHA and/or EPA), an increase in the production of endogenous anti-inflammatory lipid mediators occurs, contributing to tissue repair. Among the pro-resolution mediators produced, resolvin E1 (RvE1) is an antagonist of eicosanoid leukotriene B4 (LTB4) [60] that inhibits receptors on neutrophils, monocytes, and lymphocytes, reducing the transcription of nuclear factor κB (NFκB) and the release of tumor necrosis factor α (TNF-α) [61]. RvE1 is generated locally in response to inflammation, improving the resolution phase of inflammation, decreasing neutrophil chemotaxis, and improving the clearance of apoptotic neutrophils, directed by macrophages. It was found that RvE1 is effective in preventing and restoring bone loss in periodontitis [62,63]. Additionally, polyunsaturated fatty acids (PUFAs), such as omega-3, exert osteoprotective functions (promoting the differentiation and activation of osteoblasts), while inhibiting osteoclast activities [64]. Indeed, a very recent study reported that adjunctive omega-3 and low-dose aspirin provided clinical and immunological benefits to the treatment of periodontitis in patients with type 2 diabetes after periodontal debridement [65]. Thus, an inadequate pro-resolving response from the host may constitute a mechanism that explains the worse periodontal status in individuals with omega-3 deficiency [63].

The results of the present study also showed the participation of other nutrients, such as vitamin C (ascorbic acid), calcium, K, retinol and riboflavin. Vitamin C is necessary for collagen synthesis and also acts as an antioxidant [66]. This activity gives vitamin C a fundamental role in maintaining the integrity of connective tissues, including the periodontium [20,21,22]. Low calcium intake increases the risk of bone loss associated with periodontal disease [67,68], while low potassium intake, mainly accompanied by low fiber intake, increases blood pressure and periodontal inflammation [69]. Vitamin A plays an important role in maintaining the structural and functional integrity of mucosal cells and is necessary for the proper functioning of T and B lymphocytes and, therefore, for generating an immune response [40].

In the present study, in addition to nutritional factors, socio-behavioral factors were also associated with the periodontal status, such as age, sex, smoking, alcohol consumption, educational level, and the presence of NTCD. These results corroborate an American study in which the prevalence of periodontitis was increased due to lower levels of education, advanced age, and smoking [8]. With advancing age, essential constituents of food, such as vitamins and minerals, are absorbed less efficiently, and their production in the body decreases, thus increasing the risk of inflammatory load [19]. Still, the cells and molecules of the innate and adaptive immune response are negatively affected, including in the oral cavity, favoring periodontitis [70].

Inflammatory diseases, such as obesity, type 2 diabetes mellitus, and periodontitis induce the production of pro-inflammatory cytokines, such as Tumor Necrosis Factor alpha (TNF-α), IL-1, and IL-6, influencing the involvement and progression of each other [71]. Similarly, serum levels of total cholesterol, low density lipoprotein cholesterol (LDL-cholesterol), and triglycerides increase in the presence of periodontal disease, while periodontitis can also be a risk factor for hyperlipidemia. These findings indicate a bidirectional relationship, leading to the vicious cycle of events observed in these individuals [72].

A remarkable factor observed in our results was that 85% of the individuals with the worst periodontal status were women. Because this cluster was made up of individuals around 50 years of age, it is suggested that this result is influenced by menopause. Periodontal tissues are sensitive to hormonal changes that occur just before menopause that reduce the body’s ability to fight minor infections or maintain a balance between beneficial bacteria and pathogens in the oral environment [73]. Additionally, estrogen deficiency is associated with osteoporosis, with resulting bone loss and a predisposition to inflammatory processes [74,75].

Smoking is also known to be an important risk factor for the development of numerous systemic diseases, as well as periodontitis [76]. Smokers in various age categories exhibit significantly higher levels of antibodies to oral pathogens compared to non-smokers [77]. Smoking increases oxidative stress in periodontal tissues, inhibits defenses against biofilm bacteria, and leads to vascular constriction and slow tissue healing [78,79,80]. Smoking cessation and the consumption of antioxidant-rich foods or supplements during periodontal treatment favor tissue repair and infection control [78].

In addition to smoking, the periodontal status can be affected even more intensely by the interaction of smoking and alcohol consumption [81]. However, alcohol consumption patterns vary throughout life. Initial increases in volume during adolescence are followed by a more stable period during middle age, before the decrease in volume in older age [82]. This fact justifies the greater alcohol consumption found in the group with the best periodontal status in the present study, as it was characterized by younger individuals. Moderate alcohol intake has been reported to even provide health benefits (cardio and neuroprotection) [83]. However, excessive consumption impairs the function of T cells and neutrophils, increasing the likelihood of infection [84] and the risk of periodontitis in a dose dependent manner [85].

Finally, the present study was conducted in units of the FHS, which represents a public health program that is free and designed to be accessible to the entire population. Unfortunately, coverage is low, but the most vulnerable populations are the priority. Rural and remote areas still lack assistance. In the city where the survey was conducted, the program’s coverage rate is 56.52% (below the Brazilian average of 65.36%). However, in relation to total basic health care, the rate in the municipality is 88.78%, while for the whole country it is 76.50% [86].

The system is comprised of the registration of citizens (carried out by community agents) near the health units. These units have a family physician, a nurse, one nurse-assistant, community agents, and a dentist with one oral health assistant. An additional team composed of a nutritionist, a physiotherapist, a psychologist, and a physical education professional also support these units [87] (although some cities do not have this additional team). Approximately 50% of users live in a situation of socio-economic vulnerability [88], which was reflected in the nutritional and periodontal data. The cost of food is one of the most important determinants of food choices, and family budget constraints are barriers to the adoption of healthy choices in populations with low socioeconomic status [89,90]. Family income was not associated with periodontal status in our study because the sample comprised FHS users with similar incomes (70% had an income below two minimum wages).

Since a significant portion of this population, especially older individuals, frequently visit family health units for consultations [91], our findings indicate educational and preventive issues that might be addressed, greatly impacting the quality of life of this population. Oral health problems are directly related to socioeconomic factors, as observed in the present study. The inclusion of a dentist in the health unit seeks to modify the traditional healing–restorative philosophy in the context of primary care services. In this sense, multi-professional interactions with nutritionists, doctors, nurses, and community agents are essential for offering actual prevention and treatment measures to patients’ needs. Investing in the prevention of oral health problems, seeking low-cost measures, and employing individual and collective measures, besides gathering epidemiological knowledge, are fundamental in this context [24,92,93].

The present study has some limitations due to its cross-sectional design and population delimitation, preventing causal relationships and reducing external validity, respectively. However, cluster analysis deals with multiple variables at the same time, helping investigators to discover distinct groups in order to develop targeted programs or interventions. When designing nutritional interventions, it is crucial to know the characteristics of the intended population. In this sense, our results are particularly useful for the low-income population served by the public health system to improve dental and nutritional advice. The present results may contribute to community health promotion actions by means of encouraging nutritional campaigns and nutritional advice for specific groups of patients [30]. The identification of a nutritional profile associated with better periodontal health can be an inexpensive and safe method for the prevention and treatment not only of periodontal disease [15] in vulnerable populations assisted by the FHS, but also of other non-transmissible chronic diseases.

In summary, the results of the present study corroborate the concept that an optimized diet for oral health in low-income populations should be mainly rich in omega-3 fatty acids, fiber [16], and zinc [45]. We found that cluster 1 (poor periodontal status) participants showed lower omega-3, fiber, and Zn intakes, whereas cluster 3 (healthy periodontal status) participants showed higher omega-3, fiber, and Zn intakes. Thus, zinc, fiber, and omega-3 were the only nutrients with opposite behaviors in relation to periodontal status. This result indicates the importance of these three nutrients in order to differentiate between the two opposite clusters, 1 and 3. The relationship between nutrition and periodontal status is of paramount importance, since the loss of dental elements due to periodontitis can negatively affect the patient’s nutritional status and vice-versa, leading to the selection of soft, easy to chew, low-nutrient foods, especially in the elderly [13].

## 5. Conclusions

Diet can significantly affect periodontal status, and nutritional deficiencies are very important factors in countries with upper middle-income economies. Brazil has a public health system that greatly modifies the context of oral assistance in relation to other countries. This study highlights the influence of nutritional and sociodemographic behavioral factors on periodontal status, which may guide population-based measures for low-income populations.

## Figures and Tables

**Table 1:** Sample distribution in accordance with socio-demographic and nutritional profile (*n* = 450).

Continuous Variables	Female Mean (±SD)	Male Mean (±SD)	Range (Female/Male)
Age (years)	50 (12.5)	51.9 (13.6)	19–79/18–78
BMI	29.5 (6.1)	27.6 (5.1)	17–54.3/17.2–42.3
Energy intake (Kcal/day)	1495 (411.3)	1756 (526.3)	620–3214/772–3070
Carbohydrates (g/day)	219.26 (64.7)	248.6 (75)	89.6–475.8/108.5–437.9
Lipids (g/day)	41.6 (15.04)	48.5 (18)	12.5–92.1/10.6–102.1
Protein (g/day)	60.76 (18.5)	73.8 (25.7)	21.1–123.6/21.1–174
Zinc (mg/day)	7.41 (3.00)	9.75 (5.52)	1.9–19.6/2.4–36.5
Fiber (g/day)	21 (7.90)	25 (9.47)	3.4–46.8/7.6–44.8
Omega–3 (mg/day)	0.57 (0.21)	0.69 (0.24)	0.12–1.35/0.24–1.5
Cholesterol (mg/day)	229.5 (115.2)	278.0 (169.9)	15.5–972.1/72,6–1511.8
Calcium (mg/day)	418.3 (236.7)	445.83 (268.29)	52.2–1341.8/136.0–1295.5
Magnesium (mg/day)	195.79 (58.74)	227.33 (76.83)	66.0–402.7/77.30–451.9
Manganese (mg/day)	1.93 (0.60)	2.22 (0.76)	0.7–3.9/1.0–4.3
Phosphorus (mg/day)	892.69 (317.8)	1056.29 (395.5)	254.3–2142.8/367.2–2580.1
Iron (mg/day)	5.76 (1.89)	6.83 (2.22)	2.0–14.0/2.7–14.8
Sodium (mg/day)	1165.21 (473.16)	1334.16 (487.9)	287.3–3634.0/249.6–2902.9
Potassium (mg/day)	2193.11 (657.48)	2407.46 (787.8)	780.7–4686.0/850.8–4956.5
Copper (mg/day)	0.84 (0.44)	0.86 (0.3)	0.2–3.1/0.3–1.6
Retinol (mg/day)	170.30 (117.15)	182.02 (121.92)	4.2–779.8/19.70–654.80
Thiamine (mg/day)	0.82 (0.29)	0.92 (0.35)	0.2–2.0/0.3–2.4
Riboflavin (mg/day)	1.01 (0.52)	1.06 (0.34)	0.1–3.9/0.3–2.8
Pyridoxine (mg/day)	0.54 (0.31)	0.70 (0.50)	0.1–2.7/0–2.5
Niacin (mg/day)	12.56 (5.8)	16.89 (10.15)	1.6–45.1/2.40–58.90
Vitamin C (mg/day)	136.19 (101.63)	123.06 (101.46)	4.0–573.2/12.50–561.80
**Dichotomous variables**	**Female *n* (%)**	**Male *n* (%)**	
Presence of at least one periodontal pocket ≥ 4 mm)	234 (65.9%)	70 (73.7%)	
Physical activity practice (≥ 3×/week)	113 (32.8%)	33 (34.7%)	
Diabetes mellitus (yes)	100 (28%)	27 (28.4%)	
Hypertension (yes)	180 (50.7%)	47 (49.5%)	
Hypercholesterolemia (yes)	90 (25.4%)	16 (16.8%)	
Hypertriglyceridemia (yes)	7 (2%)	0	
Hypothyroidism (yes)	26 (7.3%)	2 (2.1%)	
Liver steatosis (yes)	7 (2%)	1 (1.1%)	
Cardiopathy (yes)	22 (6.2%)	3 (3.2%)	
Cancer history (yes)	8 (2.3%)	2 (2.1%)	
Depression (yes)	24 (6.8%)	3 (3.2%)	
Smoking (yes)	74 (20.8%)	26 (27.4%)	
Family income (>2 wages)	120 (33.8%)	33 (34.7%)	
Educational level (>8 years)	194 (54.6%)	50 (52.6%)	
Dental treatment last year	123 (34.6%)	43 (45.3%)	
Alcohol consumption (>2×/week)	29 (8.2%)	19 (20%)	

SD: Standard Deviation.

**Table 2:** Component loadings of micronutrient intake patterns obtained by principal component analysis with oblimin rotation.

Z Scores	Component		
	1	2	3
Zscore (Omega-3)	0.869		0.372
Zscore (total cholesterol)	0.701		0.525
Zscore (fiber)	0.819	−0.588	
Zscore (calcium)	0.400	−0.402	0.901
Zscore (Magnesium)	0.778	−0.699	0.456
Zscore (Manganese)	0.736	−0.660	
Zscore (Phosphorus)	0.757	−0.334	0.849
Zscore (Iron)	0.938	−0.435	0.396
Zscore (Sodium)	0.590		0.514
Zscore (Potassium)	0.684	−0.723	0.569
Zscore (Copper)	0.438	−0.764	0.309
Zscore (Zinc)	0.865		0.522
Zscore (Retinol)	0.372		0.915
Zscore (Thiamine)	0.604	−0.311	0.528
Zscore (Riboflavin)	0.320		0.848
Zscore (Piridoxine)			
Zscore (Niacin)	0.465		0.362
Zscore (vitamin C)		−0.811	

Coefficients less than 0.3 are omitted.

**Table 3:** Final cluster centers (means) of the social health-related variables (important differences which identify the clusters are shown in a dark gray color).

Variables	Cluster 1 “Poor Periodontal Status”	Cluster 2 “Intermediate Periodontal Status”	Cluster 3 “Healthy Periodontal Status”	F Test	*p*-Value
Number of cases	202	194	54		
Periodontal disease severity	1.06	0.94	0.74	3.831	0.022
Age	54.67	48.17	43.04	25.654	<0.001
Sex	0.15	0.23	0.35	5.579	0.004
BMI	29.64	28.75	28.41	1.419	0.243
Energy intake	1172.15	1706.59	2440.00	899.059	<0.001
Micronutrient intake—component 1	−0.71	0.33	1.51	240.647	<0.001
Micronutrient intake—component 2	0.31	−0.15	−0.62	22.771	<0.001
Micronutrient intake—component 3	−0.44	0.17	1.08	64.526	<0.001
Physical activity	0.34	0.33	0.31	0.132	0.876
Diabetes mellitus	0.34	0.24	0.24	2.632	0.073
Hypertension	0.55	0.50	0.35	3.576	0.029
Hypercholesterolemia	0.30	0.20	0.13	5.085	0.007
Hypertriglyceridemia	0.02	0.02	0.00	0.543	0.581
Hypothyroidism	0.07	0.07	0.02	1.007	0.366
Liver steatosis	0.03	0.00	0.02	3.433	0.033
Cardiopathy	0.05	0.07	0.04	0.487	0.615
Cancer history	0.03	0.02	0.00	0.879	0.416
Renal disease	0.00	0.01	0.00	0.137	0.872
Vascular disease	0.00	0.01	0.00	0.659	0.518
Depression	0.08	0.09	0.02	0.502	0.606
Smoking	0.28	0.19	0.13	4.194	0.016
Family income	0.34	0.38	0.27	1.071	0.344
Educational level	0.45	0.60	0.70	7.387	0.001
Dental treatment last year	0.36	0.37	0.39	0.077	0.926
Alcohol consumption	0.07	0.11	0.20	3.829	0.022

BMI, body mass index. “Female sex” and “Yes” answers were set as = 1.

## Data Availability

Data will be available upon request.
